# Lingual mucosal graft treatment for recurrent renal bleeding after ureteral stricture surgery: A case report

**DOI:** 10.1097/MD.0000000000042384

**Published:** 2025-09-05

**Authors:** Longyuhe Yang, Yueqiang Wang, Jianbing Yang, Yunliang Zhao, Zhen Ma, Zhigang Zhang

**Affiliations:** a Urology Department, Southern Central Hospital of Yunnan Province (First People’s Hospital of Honghe State), Mengzi, Yunnan Province, China; b Anesthesiology Department, Southern Central Hospital of Yunnan Province (First People’s Hospital of Honghe State), Mengzi, Yunnan Province, China.

**Keywords:** lingual mucosal graft, renal hemorrhage, repair and reconstruction, ureteral stricture

## Abstract

**Rationale:**

Ureteral stricture is a complex urological condition often requiring surgical intervention. Autologous tissue grafts, such as lingual mucosa, have emerged as a promising option for reconstruction due to their favorable biocompatibility and vascularity. However, reports on complications associated with these techniques remain limited.

**Patient concerns:**

A 59-year-old male with a history of bilateral kidney stones, chronic renal failure, hypertension, and multiple prior ureteral surgeries presented with left ureteral obstruction and severe hydronephrosis. He had been on regular dialysis and previously underwent several endoscopic and percutaneous stone procedures.

**Diagnoses:**

Preoperative imaging confirmed a long-segment stricture in the upper left ureter accompanied by significant hydronephrosis and impaired renal function.

**Interventions:**

The patient underwent laparoscopic resection of the strictured segment followed by ureteroplasty using an autologous lingual mucosal graft. A double-J stent was placed, and the graft was secured with absorbable sutures and wrapped with omentum.

**Outcomes:**

Postoperatively, the patient experienced 2 episodes of renal hemorrhage, likely exacerbated by anticoagulation during dialysis and underlying vascular fragility. After multidisciplinary management, including cessation of heparinized dialysis and transition to non-anticoagulant therapies, the bleeding resolved. Three-month follow-up showed patent ureter, resolved hydronephrosis, stable renal function, and no recurrence of symptoms.

**Lessons:**

Lingual mucosal graft ureteroplasty is a viable option for complex ureteral strictures, but careful patient selection is critical – particularly in those with chronic renal failure on dialysis. Intraoperative techniques to avoid renal pelvic injury and judicious postoperative anticoagulation management are essential to prevent hemorrhage. A multidisciplinary approach and close follow-up are key to successful outcomes.

## 1. Introduction

Ureteral stricture (US) is a common urological condition characterized by narrowing of the ureteral lumen due to various causes, which subsequently affects urine drainage and significantly affects the patient’s quality of life and renal function. Clinically, the causes of US are diverse, with common factors including congenital malformations, trauma, postoperative scar tissue, infections, and tumors. This condition typically presents as hydronephrosis, impaired renal function, or even renal failure, making timely and effective treatment particularly crucial.^[1]^

Currently, the treatment methods for US can be divided into 2 main categories: endoscopic and non-endoscopic. Endoscopic treatments typically include stent placement, stricture incision, and balloon dilation, which can achieve better results in patients with mild strictures.^[2]^ However, the efficacy of these methods is often limited by the degree and location of the stricture and maintaining long-term patency can be challenging.

Non-endoscopic surgical treatment is an important option for more severe or complex cases. Traditional surgical methods include end-to-end anastomosis after resection of the narrowed segment of the ureter, and ureteral reimplantation into the bladder. Although these surgeries can restore urinary flow to some extent, in patients with longer segments or multiple narrowed segments, tension after complete resection of the narrowed segment may be relatively high, potentially leading to the development of new strictures and resulting in surgical failure.

In recent years, autologous tissue patches haves emerged as a new therapeutic strategy. This approach offers promising results for US treatment. Autologous lingual mucosa patches have been increasingly applied in the repair of USs owing to their rich blood supply and good biocompatibility. This approach not only effectively avoids rejection reactions that may arise from allogeneic grafts but also exhibits favorable healing characteristics.^[3]^

## 2. Case report/case presentation

### 2.1. Basic information

This case involved a 59-year-old male patient who was admitted on May 25, 2023, because of left ureteral stones and severe hydronephrosis of the left kidney. His medical history included “bilateral kidney stones and chronic renal failure” for over 2 years, during which he underwent multiple treatments at other hospitals, including “bilateral percutaneous nephrolithotomy (PCNL) and ureteroscopic lithotripsy (URSL) procedures.” In February 2021, the patient underwent L-URSL and B-URSL in March 2022, L-URSL in June 2022, and L-PCNL in January 2023. He had a history of hypertension and chronic renal failure and had undergone regular dialysis. The patient also had a history of blood transfusions. computed tomography (CT) upon admission revealed stones in the upper segment of the left ureter, accompanied by significant hydronephrosis, with a serum creatinine level of 541.4 μmol/L. After comprehensive examination, the patient underwent L-PCNL at our hospital. During the procedure, a stricture was noted in the upper segment of the left ureter, and 2 double-J stents (F5) were placed for dilation. Three months later, the stents were removed, and dialysis treatment was continued locally. One week after stent removal, the patient reported discomfort and mild pain in the left flank accompanied by fever. This raised the suspicion of a left renal abscess infection, prompting a return to the hospital for left renal puncture and drainage, yielding 400 to 600 mL of drainage fluid daily. Postoperatively, left renal stent imaging and retrograde pyelography revealed a stricture in the upper segment of the ureter.

### 2.2. Surgical method

**Figure 1.**
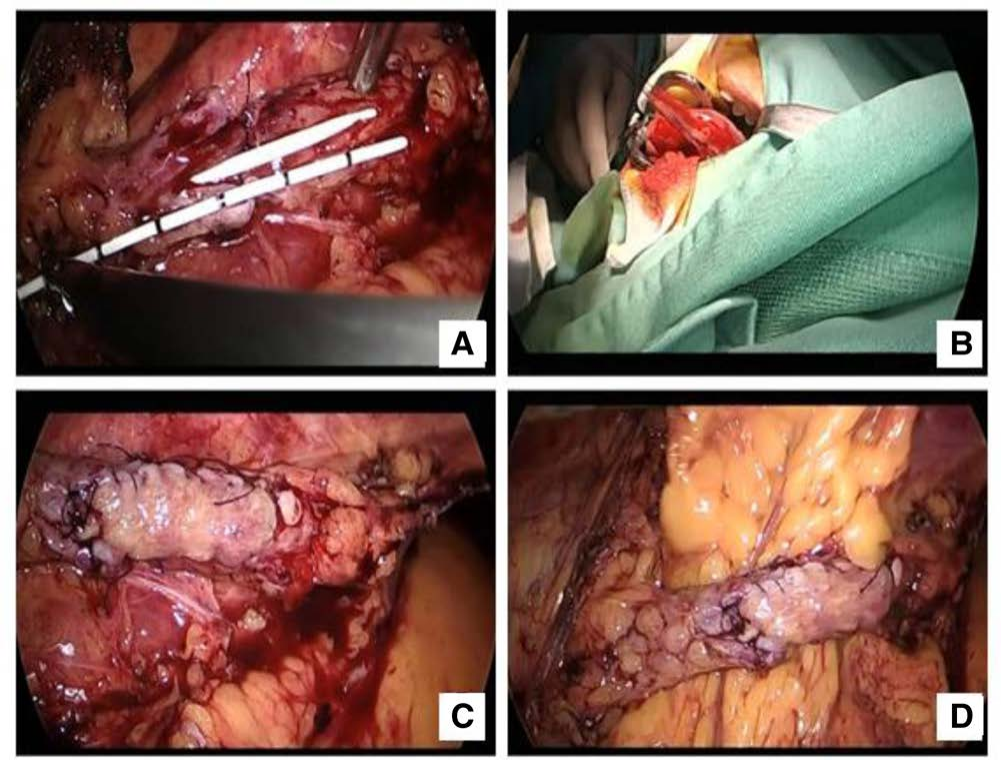
Surgical procedure of laparoscopic tongue mucosa patch formation. (A) Freeing the narrowed segment of the ureter and resection, followed by posterior wall reinforcement suturing and measurement of the defect length. (B) Measuring an appropriate length of oral mucosa, cutting it, and placing it in ice water for later use. (C) Continuously suturing the tongue mucosa patch to the ureter. (D) Wrapping the ureter with omentum to provide protection and a certain degree of blood supply to the tongue mucosa.

After fully explaining the condition to the patient and their family, consent was obtained for laparoscopic US resection with end-to-end anastomosis (oral mucosa grafting). Two days before surgery, a chlorhexidine mouthwash was used for oral care. After anesthesia was administered, the patient was placed in a lithotomy position. Under guidewire assistance, the left ureter was accessed, and upon entering the ureteroscope 20 cm, the lumen was pinhole-sized with stiff tissue, preventing passage of the ureteroscope. A ureteral catheter was inserted and the scope was withdrawn, leaving an F18 3-lumen catheter in place. The patient was then positioned in the left lateral decubitus position, and a veress needle was inserted at the umbilicus to establish pneumoperitoneum. The skin was incised 1.5 cm along the left rectus abdominis at the umbilicus, and a 10 mm trocar was used to create an access port. A laparoscope was introduced into the abdominal cavity, and no abnormalities were observed. A 1.0 cm skin incision was made 2 cm below the left clavicle along the midclavicular line, and an 8 mm trocar was inserted into the left hand. A 1.5 cm skin incision was made along the anterior axillary line at the level of the umbilicus, and a 12 mm trocar was inserted into the right hand. An ultrasonic scalpel was used to dissect the peritoneum adjacent to the ascending colon, exposing the US site. Significant adhesions were noted around the stricture, which were released using an ultrasonic scalpel approximately 5 cm from the ureteropelvic junction. The stricture segment of the ureter was cut with scissors, measuring approximately 2 cm in length. However, the ureteral tissue above and below the stricture was stiff, resulting in a longer defect after resection. Therefore, we decided to perform ureteroplasty using an oral mucosal graft. The catheter was removed, and the ureter was reinforced with 4-0 absorbable sutures on the posterior wall (Fig. 1A). A double-J stent (F6) was inserted to measure the ureteral defect to be 4 cm long. The oral cavity was disinfected, and 3-0 absorbable sutures were used to suture the outer 2 stitches of the left lingual mucosa. A segment of the left lingual mucosa measuring 4.5 cm in length and 1.0 cm in width was marked. Saline was injected submucosally, and the marked mucosa was excised. After trimming, the graft was soaked in saline at 4 °C, and the mucosal wound was continuously sutured with 5-0 absorbable sutures (Fig. 1B). The lingual mucosa was then placed into the abdominal cavity and continuously sutured to the ureteral defect using 4-0 absorbable sutures (Fig. 1C). The omentum was wrapped around the ureteroplasty site, and 3-0 barbed sutures were used to secure it (Fig. 1D). No significant active bleeding was observed, and a drainage tube was placed in the abdominal cavity at the ureteroplasty site. All trocars were removed, and the drainage tube was secured with sutures, followed by closure of all incisions. Approximately 200 mL of blood was lost and the surgery was successfully completed.

### 2.3. First kidney hemorrhage

**Figure 2.**
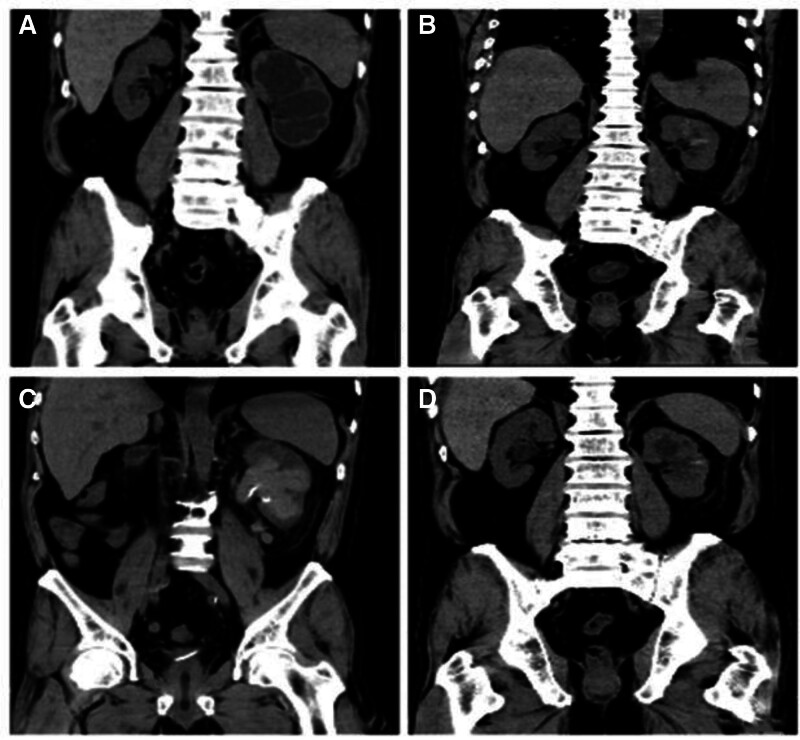
CT changes before and after surgery. (A) Preoperative moderate to severe hydronephrosis of the left kidney. (B) Hemorrhage in the left renal pelvis postoperatively. (C) Significant hematoma in the left renal pelvis after heparinized dialysis. (D) Hematoma in the left renal pelvis has resolved spontaneously before discharge. CT = computed tomography.

On postoperative day post-surgery, the drainage fluid was red. A follow-up CT scan indicated hematoma in the left renal pelvis (Fig. 2B). During surgery, the injection of saline through the stoma to locate the narrowed ureter caused mucosal bleeding in the left renal pelvis. Hemostatic medications were administered, and anti-infection treatment was intensified. The patient’s creatinine level increased postoperatively. Due to regular dialysis prior to surgery, heparinized dialysis was performed on the fourth postoperative day. After dialysis, the color of the stoma and ureter drainage deepened, but the was no significant decrease in the hemoglobin level. Hemostatic treatment was continued, and urine color gradually decreased. On the eighth postoperative day, heparinized dialysis was performed, and the drainage fluid from the stoma and ureter turned bright red. A follow-up CT scan revealed a large blood clot in the left renal pelvis (Fig. 2C), and 2 units of suspended red blood cells were transfused the following day. On the 15th postoperative day, no significant bleeding was observed, and a follow-up CT scan indicated that the blood clot in the left renal pelvis had dissolved on its own (Fig. 2D). The infection markers were normal, vital signs were stable, and the patient was discharged after the removal of the urinary catheter.

### 2.4. Second renal hemorrhage

**Figure 3.**
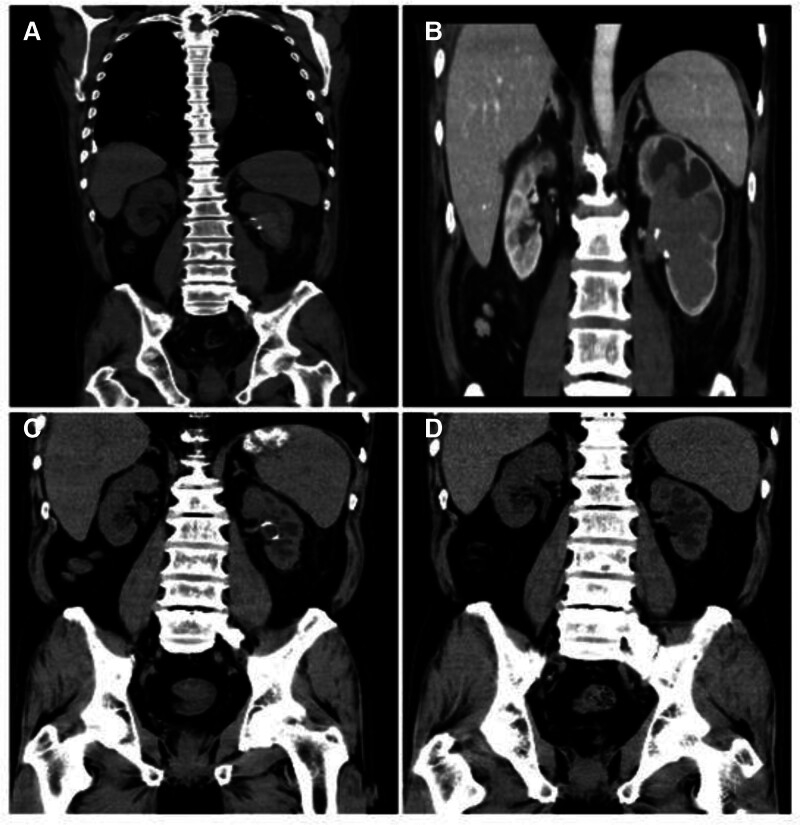
CT changes after the second renal hemorrhage. (A) Significant hematoma in the left renal pelvis after heparinized dialysis at an external hospital. (B) After non-heparinized dialysis, the left kidney showed no arteriovenous fistula or pseudoaneurysm formation on CT enhancement. (C) Hematoma in the left renal pelvis resolved spontaneously after dialysis was stopped. (D) Four months post-surgery, removal of the double-J stent showed no significant worsening of left renal hydronephrosis. CT = computed tomography.

One week after discharge, drainage fluid from the left kidney stoma was clear. The patient visited a local hospital for hemodialysis once, and on the same day, the stoma tube turned bright red, with an inability to urinate, leading to an emergency transfer to our hospital. A CT scan upon admission indicated hematoma in the left renal pelvis (Fig. 3A). Examination results revealed a creatinine level of 1023.4 μmol/L, and a potassium level of 6.9 mmol/L, suggesting reduced blood volume and inadequate perfusion. An emergency blood transfusion was performed, followed by hemodialysis, anti-infection treatment, urinary catheter placement, and continuous bladder irrigation. Blood transfusion therapy was continued the following day. Subsequently, 2 sessions of heparin-free dialysis were maintained, with no significant bleeding observed, and the stoma and urinary catheter remained clear. However, after the fourth heparin-free dialysis session, the stoma and urinary catheter turned bright red again, with a progressive decline in hemoglobin levels. Arteriovenous fistula or pseudoaneurysm formation was suspected, and CT angiography did not reveal any clear vascular bleeding points (Fig. 3B). Hemorrhage from the renal mucosa was still considered, and the medication for hemostasis was continued. An investigation of the coagulation factors revealed no abnormalities.

After multidisciplinary discussion, it is recommended to discontinue hemodialysis and switch to oral uremic-clearing granules, sodium bicarbonate, sodium zirconium silicate, rosuvastatin, or compound α-keto acid treatment. Subsequent CT reexamination showed that the left renal pelvis hematoma had resolved (Fig. 3C), and the left nephrostomy tube was removed.

## 3. Results

After 3 weeks of hospitalization, the patient was successfully discharged. Following discharge, no further hemodialysis treatment was administered. Three months later, follow-up CT showed that the left ureter was unobstructed, hydronephrosis had resolved, and the symptoms had completely resolved. The left ureteral stent was removed. At the 1-month post-removal follow-up, there was no worsening of the left hydronephrosis (Fig. 3D), and the left flank pain had completely resolved. Daily urine output was maintained at 1000 to 1500 mL, and creatinine levels were stable at 530 to 600 μmol/L. The patient was advised to resume hemodialysis; however, due to concerns about potential rebleeding, he declined treatment and continued with regular outpatient follow-ups every 3 months.

## 4. Discussion

### 4.1. Selection of indications

The oral mucosa is smooth and flat, often exposed to moist conditions for extended periods, and is characterized by thick epithelial tissue, good elasticity, thin lamina propria, and abundant capillaries. These features facilitate the revascularization of the transplanted tissue, making it an ideal graft material. After repairing the narrowed areas of the ureter with tissue grafting, the survival rate of the transplanted tissue was high, with no complications such as retraction, necrosis, or urinary leakage.

Histological results indicated a significant trend toward urothelialization in the epithelium posttransplant, accompanied by a substantial presence of newly formed capillaries in the mucosa following the grafting procedure. Therefore, oral mucosal graft techniques haves promising applications in urological surgeries.^[4]^

However, oral mucosal grafts for US still have certain limitations, such as donor site trauma and postoperative wound healing, which should attract the attention of physicians.^[5]^

Currently, the indications for the use of autologous lingual mucosal grafts are not clearly defined and should be assessed comprehensively based on the specific circumstances of the patient. When selecting indications, several aspects need to be considered: The location, length, and nature of the stricture. For longer or more complex USs, performing traditional end-to-end anastomosis after resecting the stricture segment can result in excessive tension, leading to a potential risk of postoperative restenosis. Autologous lingual mucosa grafts effectively address this issue by providing better repair outcomes without tension on the ureter.^[6,7]^ The overall health status of the patient and any comorbidities must also be considered. In this case, we learned during the preoperative evaluation that the patient had a history of hemodialysis. Renal function of the affected side due to US was evaluated solely based on the daily drainage volume after nephrostomy, without considering the function of the unilateral kidney. The appropriateness of renogram as a routine preoperative assessment standard for US surgery warrants further investigation. The patient’s symptoms were completely relieved postoperatively, and complications associated with long-term nephrostomy tube use were avoided. However, repeated treatments for bleeding introduced a certain economic burden and damage to the patient. This also serves as a reminder that surgical options should be chosen with caution in patients with severe underlying diseases or poor postoperative recovery. Patients’ willingness and understanding of surgical risks should be prioritized. Adequate preoperative communication can help patients make informed decisions. In this case, the patient demonstrated a strong desire to undergo surgery and insisted on proceeding, even after being informed of potential complications. After experiencing related complications postoperatively, the patient was cooperative in treatment, and mutual trust and communication between the medical staff and patient were crucial factors for the successful execution of such surgeries.

### 4.2. Analysis of causes of renal hemorrhage

In this case, the patient experienced recurrent renal hemorrhage after autologous lingual mucosal patch repair. Renal hemorrhage may be caused by multiple factors. Intraoperative injury was a significant contributing factor. While searching for a proximal US, it is common to confirm its location by infusing saline into the renal nephrostomy tube to identify the dilated segment. However, we used a method of pushing saline through the nephrostomy tube, which resulted in an excessively high pressure within the renal pelvis in a short period. Coupled with the kidney’s poor contraction function and increased vascular fragility, this led to rupture of the surrounding blood vessels in the renal pelvis, causing postoperative bleeding. Currently, for the localization of US segments, indocyanine green near-infrared fluorescence imaging can accurately highlight the location of the US.^[8]^ This technique has been effectively applied in laparoscopic US repair and reconstruction surgery. In future US reconstruction surgeries, it is essential to remind surgeons to consider using fluorescent agents to locate USs, thereby avoiding similar injuries.

Second, postoperative infections are potential causes of bleeding. Based on the results of the infection indicators reviewed after each bleeding episode, procalcitonin levels showed a significant increase. Urinary tract infections may trigger local inflammatory responses, leading to congestion, edema, and even erosion of the renal pelvis mucosa, which in turn affects the healing process and increases the risk of bleeding. Additionally, increased vascular permeability allows red blood cells to leak, resulting in recurrent bleeding. Therefore, in patients undergoing US repair with autologous patch grafts, it is essential to ensure infection control before surgery when carrying a nephrostomy tube for an extended period.

Postoperatively, routine follow-up should include blood counts, infection indicators, and urine cultures, with antibiotic use adjusted according to drug sensitivity to ensure graft viability and prevent local infections that could lead to restenosis, thereby improving the success rate of surgery.^[9]^

Finally, the patient’s underlying conditions, anticoagulation therapy, and postoperative activity levels may also influence the occurrence of bleeding. In the present case, the patient had a long history of hemodialysis. Although coagulation function and coagulation factors appear normal, the possibility of intrinsic coagulation abnormalities causing renal bleeding cannot be ruled out.

### 4.3. Discussion on the timing of postoperative dialysis

In the present case, the patient experienced renal hemorrhage after surgery, leading to hypovolemia and a sharp increase in renal function. However, varying degrees of renal hemorrhage occurred after each dialysis session, further increasing renal function. Therefore, the timing of dialysis is crucial. Considering the causes of bleeding in other areas of hemodialysis patients,^[10]^ we believe that recurrent renal bleeding post-dialysis may involve the following mechanisms: frequent use of anticoagulants increases the risk of bleeding; chronic kidney disease may cause hemodynamic instability in renal blood supply, hindering the healing of the renal pelvis mucosa; and platelet dysfunction, interactions between platelets and the vascular wall, and coagulation abnormalities.

Generally, indications for dialysis include severe electrolyte disturbances, uremic symptoms, and acute renal failure. When assessing the timing of dialysis, it is essential to closely monitor the patient’s renal function indicators, such as blood urea nitrogen, creatinine levels, and electrolyte status. If there are clear signs of renal failure, dialysis should be promptly performed to avoid further complications.^[11]^ Early postoperative recovery of renal function should also be considered. If the patient’s renal function improves shortly after surgery, dialysis can be delayed. Additionally, using Nafamostat for anticoagulation treatment in patients with a high risk of bleeding during hemodialysis can effectively improve the patient’s dialysis situation, regulate electrolyte levels and coagulation function, enhance dialysis adequacy, and have a minimal impact on nutritional status, demonstrating favorable application effects.^[12]^

### 4.4. Follow-up considerations

For patients undergoing autologous oral mucosal graft repair surgery, postoperative follow-up is a key component to ensure long-term effectiveness and safety. Follow-up should include regular monitoring of renal function, imaging examinations, and assessment of clinical symptoms. Specifically, renal function evaluations should be conducted at 1, 3, and 6 months post-surgery, with ultrasound or CT scans performed as necessary to assess the patency of the ureters.

Additionally, it is important to monitor for signs of complications such as infection and bleeding, and promptly address these issues. Furthermore, the patient’s psychological state and quality of life should be included in the follow-up scope, with psychological support and rehabilitation guidance provided as needed.

## Author contributions

**Conceptualization:** Yueqiang Wang.

**Data curation:** Jianbing Yang.

**Formal analysis:** Jianbing Yang.

**Investigation:** Yunliang Zhao.

**Resources:** Zhen Ma.

**Supervision:** Zhigang Zhang.

**Writing – original draft:** Longyuhe Yang.

**Writing – review & editing:** Longyuhe Yang.
